# Severe scleroderma colopathy at initial diagnosis of systemic sclerosis in a young man: a case report

**DOI:** 10.3389/fmed.2026.1784858

**Published:** 2026-03-12

**Authors:** Murad Isaak Alshamisti, Nouraldeen Deeb, Ahmad Almasalmah, Samer Amayre, Issa Abu Iram, Saed Ismael Atawnah, Mohammad Ibriwesh

**Affiliations:** 1 Faculty of Medicine, Al-Quds University, Jerusalem, Palestine; 2 Internal Medicine Department, Al-Ahli Hospital, Hebron, Palestine

**Keywords:** case report, colonic dysmotility, gastrointestinal involvement, malnutrition, scleroderma, systemic sclerosis

## Abstract

Scleroderma, or systemic sclerosis (SSc) usually affects the gastrointestinal (GI) tract (up to ~90%), but severe lower GI dysmotility and intestinal pseudo- obstruction are uncommon, occurring in approximately 5. 4% of patients with SSc. We report a 25-year-old Palestinian male with a three- year history of profound unintentional weight loss (67 kg) and an eight- month history of postprandial diffuse abdominal pain, bloating, vomiting, diarrhea, and loss of appetite. Examination revealed cachexia, digital ulcers, and generalized skin thickening with hyperpigmentation. Inflammatory markers were elevated (CRP/ESR) with positive antinuclear antibody. Upper endoscopy demonstrated severe reflux esophagitis extending to the upper esophagus and mild erosive gastritis of the antrum. Colonoscopy revealed marked colonic hypomotility with minimal peristalsis and retained fecal matter. The patient was treated with mycophenolate mofetil beside symptomatic and supportive therapy (rifaximin, hyoscine butylbromide, and metoclopramide), with clinical improvement on follow-up. This case highlights severe SSc- related lower- GI dysmotility in a young male with extreme weight loss and chronic GI symptoms. Clinicians should consider SSc in patients with unexplained dysmotility/pseudo-obstruction features, especially when associated with Raynaud- spectrum vascular features, skin thickening, or positive ANA to reduce diagnostic delay and prevent further consequences, such as malnutrition.

## Introduction

Systemic sclerosis (SSc) is a multisystem autoimmune connective tissue disease characterized by immune dysregulation, microvascular injury, and progressive fibrosis of the skin and internal organs. It shows a marked female predominance (female-to-male ratio 4–6:1), most commonly begins in mid- to late adulthood; early clinical clues include Raynaud's phenomenon with digital color changes upon cold exposure and progressive thickening of the fingers (puffy fingers, sclerodactyly), which may precede visceral involvement by months to years (1). SSc is classified using the 2013 EULAR/ACR criteria (2).

The gastrointestinal (GI) tract is the most frequently affected internal organ system after the skin, with >90% of patients developing some degree of GI involvement, including gastroesophageal reflux, esophageal dysmotility with dysphagia, small-intestinal bacterial overgrowth, and colonic dysmotility (3). Importantly, severe GI disease may occur early in the disease course and was associated with higher mortality and poorer health-related quality of life (4). Malnutrition is common and tightly linked to GI involvement and advanced disease severity (5).

Within this context, chronic intestinal pseudo-obstruction (CIPO) and severe lower-GI dysmotility are uncommon but clinically consequential. In a nationwide U.S. inpatient analysis of 193,610 SSc hospitalizations, intestinal pseudo-obstruction occurred in 5.4% of SSc hospitalizations and was associated with prolonged hospital stays, greater need for total parenteral nutrition, and an in-hospital mortality rate of 7.3% (6). Individual case reports further highlight severe presentations of SSc-related CIPO and the substantial challenges associated with intestinal failure, complementing population-level data (7). Here, we report a 25-year-old male with newly diagnosed SSc who presented with profound unintentional weight loss (67 kg over three years), chronic abdominal pain and bloating, and colonoscopic evidence of an akinetic colon with retained fecal matter consistent with scleroderma colopathy in the absence of mechanical obstruction. This case highlights the potential for profound and early lower gastrointestinal involvement in SSc, emphasizing the need for heightened clinical suspicion and early nutritional assessment even at initial presentation.

## Case presentation

A 25-year-old Palestinian male presented to the outpatient clinic with an 8-month history of diffuse postprandial abdominal pain associated with bloating, vomiting, diarrhea and anorexia. He reported progressive fatigue and profound unintentional weight loss of 67 kg over three years (120 kg in 2022–53 kg in 2025). He additionally described episodic cold-induce bluish discoloration of the fingers of his hands resolved with warming progressive diffuse skin hyperpigmentation beginning on the back and subsequently involving the abdomen, arms, and the face. He denied recent infections, known allergies, chronic medical illnesses, or a family history of autoimmune diseases. He was not taking any regular medications. The patient worked as a construction worker and smoked (≈5 pack-years).

**Figure 1:**
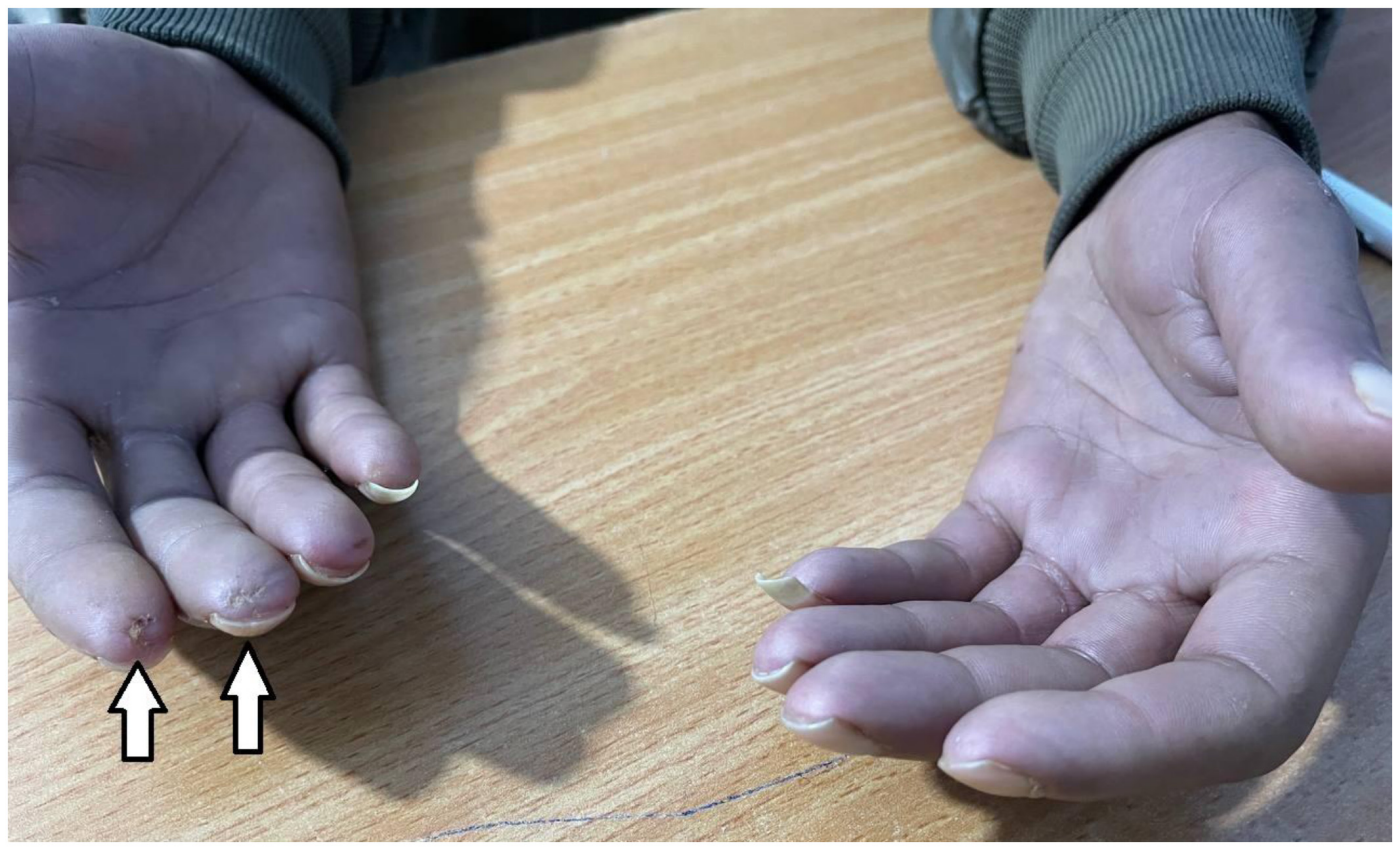
Digital ulcers involving the index and middle fingers of the right hand (arrows). The ulcers demonstrate loss of skin integrity with an exposed ulcer base covered by necrotic tissue. The surrounding skin appears tight and shiny, with dusky discoloration. Findings are consistent with ischemia related to systemic sclerosis.

On physical examination, he appeared pale and cachectic. Vital signs were stable (BP 110/75 mmHg, HR 84 bpm, RR 17/min, SpO2 95% room air, T 36.8 °C). Facial examination demonstrated microstomia (“fish-mouth” appearance) with limited mouth opening. Cutaneous examination revealed skin thickening of the fingers of both hands extending proximal to the metacarpophalangeal (MCP) joints, with ulcerations at the tips of the digits and associated bluish discoloration (Figure 1). Diffuse skin hyperpigmentation was observed over the back, abdomen, arms and face. Musculoskeletal examination showed restricted active motion of MCP, proximal interphalangeal (PIP) and distal interphalangeal (DIP) joints.

Laboratory investigations demonstrated elevated erythrocyte sedimentation rate (ESR) and C-reactive protein (CRP). Autoimmune testing revealed positive antinuclear antibodies (ANA), while Extractable nuclear antigens (ENA) and anti-Scl-70 antibodies were negative (Table 1). Endocrine evaluation showed normal thyroid-stimulating hormone (TSH) and early morning cortisol levels. Renal function tests, including serum creatinine and blood urea nitrogen (BUN), were within normal limits. Liver profile revealed hypoalbuminemia with normal alanine aminotransferase (ALT) levels. Cardiovascular evaluation (ECG, echocardiography and Doppler studies) was unremarkable.

**Table 1:** Timeline table.

**Date/period**	**Clinical course**	**Work up/key results/management**
~September 2022	Noticed minimal weight loss (Baseline ~ 120 kg).	—
Oct 2022–Feb 2023	Further weight loss (~9 kg). Progressive loss of Appetite. Intermittent minimal diffuse abdominal pain, and postprandial bloating. Mild fatigue.	—
Mar 2023	Bluish discoloration of digits on cold exposure (relieved by warming). Gradual hyperpigmentation first noticed on the back.	—
Nov 2023	Hyperpigmentation spread to abdomen, arms, and face.	—
Jan 2024–Aug 2025	Marked progressive weight loss (total 67 kg). Progressive limitation of hand and finger movement. Worsening generalized fatigue. Worsening gastrointestinal symptoms.	—
27 Aug 2025 (GI visit)	Evaluated for weight loss.	Tests performed: • CBC (Hb 13) • Serum Albumin 2.9 • TSH 2.25 • Brucella: negative • Anti-tTG 4.9 • Upper endoscopy: severe reflux esophagitis extending to upper esophagus; mild erosive gastritis • Colonscopy: sigmoid full of stool; difficult to clean.
23 Oct 2025 (Rheumatology - 1^st^ visit)	History and examination performed.	Initial labs: • CBC (Hb 13, WBC 10,800, PLT 560^*^1000) • AST 18, ALT 18 • Creatinine 0.7 • CRP 24, ESR 20 • Cardiac evaluation: • Echocardiography: EF 57%, otherwise normal. • Plan: • ANA, ENA • Early-morning cortisol level • Colonscopy • Symptomatic meds: • Flatex 20 mg PRN • Scobutyl 10 mg (1^*^2) • Follow- up: 2 weeks
6 Nov 2025 (Rheumatology follow- up)	—	Results: • ANA 1:320 • ENA negative • Early- morning cortisol 14.1 • Colonscopy: • Akinetic colon (image consistent with scleroderma colopathy) • Plan/treatment: • Cellcept 500 mg (1^*^2) • Rifaximin 500 mg (1^*^3) ^*^10 days • Scobutyl 10 mg (1^*^2) ^*^10 days • Pramin 10 mg (1^*^2) ^*^10 days • Follow- up: 1 month
4 Dec 2025 (Follow- up)	Symptoms improved markedly after therapy. Weight regain (+1 kg over 1 month)	Continue prescribed medications. Follow- up: 1 month

ANA, antinuclear antibody; ENA, extractable nuclear antigen; CRP, C- reactive protein; ESR, erythrocyte sedimentation rate; EF, ejection fraction; tTG, tissue transglutaminase; PRN, as needed.

Upper gastrointestinal endoscopy demonstrated severe reflux esophagitis extending to the upper esophagus and mild erosive gastritis in the antrum. Colonoscopy revealed no obstructing lesion but demonstrated marked colonic hypomotility with minimal peristalsis and retained fecal matter, consistent with scleroderma- associated colopathy (Table 1). The patient met the 2013 EULAR/ACR classification criteria for systemic sclerosis (total score 15; threshold ≥9).

The patient was initiated on mycophenolate mofetil (CellCept) 500 mg orally twice daily, plus rifaximin, hyoscine butylbromide, and metoclopramide for symptomatic management. At one- month follow-up, he reported marked improvement in GI symptoms with early weight regain (~1 kg over one month).

Written informed consent for publication of this case and accompanying image was taken from the patient.

## Discussion

Gastrointestinal (GI) involvement is among the most frequent and clinically burdensome manifestations of systemic sclerosis (SSc), affecting a substantial proportion of patients over the disease course and contributing meaningfully to impaired quality of life, malnutrition, hospitalization, and mortality (1, 3). The present case is notable for severe, predominantly lower-GI manifestations—marked weight loss with hypoalbuminemia, alternating diarrhea/constipation, and colonoscopic evidence of profound colonic hypomotility with stool retention—occurring in a young adult male and preceding/overshadowing other systemic features. This pattern reinforces that significant GI disease may occur early in SSc and can be a defining feature at presentation rather than a late complication (4).

### Diagnostic challenges

Systemic sclerosis was not initially suspected given the patient's young age and male sex.

The presentation was dominated by severe GI symptoms and profound weight loss, broadening the differential diagnosis. Severe colonic dysmotility/pseudo-obstruction can mimic mechanical obstruction; colonoscopy showed marked hypomotility with stool retention but no structural lesion. Serology was not “classic” (ANA positive with negative ENA/anti–Scl-70), so diagnosis relied on the overall clinical picture and EULAR/ACR criteria.

### Pathophysiology and diagnostic considerations

SSc-related GI dysfunction is multifactorial, reflecting a combination of neurogenic impairment, smooth muscle atrophy, vascular dysfunction, and progressive fibrosis, which together impair coordinated motility across the GI tract (1, 3, 8, 9). Stasis promotes dysbiosis and small intestinal bacterial overgrowth (SIBO), which can manifest with bloating, abdominal pain, diarrhea, and malabsorption and may accelerate nutritional decline (8–11). In our patient, severe weight loss (with low serum albumin) alongside mixed bowel habits raised concern for malabsorption and/or intestinal failure physiology in addition to primary dysmotility. While colonoscopy showed grossly normal mucosa without mechanical obstruction, the marked hypomotility and retained fecal matter supported a motility disorder consistent with SSc colopathy and/or pseudo-obstruction-spectrum disease.

Clinically, intestinal pseudo-obstruction in SSc represents a severe dysmotility phenotype characterized by obstructive symptoms in the absence of a fixed mechanical lesion (6, 7). Because symptoms and signs overlap with true obstruction, an essential step is to exclude structural causes (e.g., strictures, malignancy, volvulus), medication-related ileus, metabolic disturbances, and infectious etiologies before attributing symptoms to primary dysmotility (8, 9). The negative anti-tTG IgA and normal cortisol in this case also helped reduce the likelihood of alternative explanations for profound weight loss such as celiac disease or adrenal insufficiency, respectively. Importantly, severe GI disease in “very early” SSc has been associated with worse outcomes and early mortality, making prompt recognition and supportive management crucial even when other organ involvement is not yet advanced (4).

### Therapeutic considerations and clinical course

Current management of SSc-GI dysmotility is largely supportive and symptom-directed because disease-modifying options for established dysmotility remain limited (8, 10, 12). In our patient, a pragmatic regimen including a prokinetic (metoclopramide), antispasmodic therapy, and rifaximin for presumed bacterial overgrowth was associated with symptomatic improvement on follow-up, supporting a clinically meaningful, treatable component related to dysmotility-associated stasis/SIBO rather than irreversible end-stage fibrosis alone. Evidence for antibiotic treatment in SSc-associated SIBO is heterogeneous but suggests that antibiotics can eradicate SIBO in a subset of patients, although recurrence is common and repeated courses may be required (11, 13, 14). For refractory pseudo-obstruction-spectrum disease, small studies and physiological trials support consideration of octreotide to stimulate intestinal motility and reduce bacterial overgrowth, and selected patients may benefit from newer prokinetics such as prucalopride, but data remain limited (14, 15, 23).

### Diagnostic emphasis and clinical implications

This case highlights that severe unintentional weight loss with bloating, diarrhea, and vomiting in a young patient may be consistent with severe SSc-related intestinal dysmotility with SIBO and psudo-obstruction-spectrum disease, rather than a primary GI diagnosis. GI involvement is comon in SSc and small bowel hypomotility can lead to malabsorption and nutritional failure through bacterial overgrowth and transit delay. In clinical practice, the distinguishing feature is between: (1) mechanical obstruction or other systemic causes of profound weight loss and (2) functional obstruction from dysmotility; assessment should therefore prioritize exclusion of structural obstruction (imaging/endoscopy as indicated) without compromising early suspicion for dysmotility/SIBO when symptoms cluster around postprandial worsening, bloating, and fluctuating bowel habits in patient with SSc manifestations. Early recognition matters because rapid symptom-based treatment and nutritional escalation can be clinically meaningful even when fibrosis is present (11, 16, 17).

### Nutritional implications

Malnutrition and nutrition-related conditions are increasingly recognized as underappreciated but clinically important problems in SSc (5). GI dysmotility, bacterial overgrowth, reduced intake (from pain, bloating, reflux), and systemic inflammation can converge to produce rapid nutritional decline, as reflected in this case by major weight loss and hypoalbuminemia. Early nutrition assessment (including weight trajectory, BMI, micronutrient evaluation, and functional status) is therefore central to SSc-GI care (5, 8, 12). In advanced intestinal failure related to SSc, home parenteral nutrition (HPN) has been reported as a feasible long-term strategy to preserve nutritional status when oral/enteral routes are inadequate (17). Even when HPN is not required, proactive dietary strategies (small frequent meals, texture modification, targeted supplementation, and management of reflux) and prompt treatment of SIBO can be decisive in stabilizing weight and improving symptoms (8, 12, 14).

### Review of previously reported cases

Severe lower-GI complications in SSc are well described but are reported disproportionately in older patients and in women, reflecting the underlying epidemiology of SSc. A systematic review focused on colonic manifestations and complications highlighted that constipation and diarrhea can coexist and that serious outcomes—including pseudo-obstruction, fecal impaction, megacolon, volvulus, ischemia, and perforation—occur across reported cases (18). In parallel, population-level data suggest that pseudo-obstruction is an important cause of hospitalization in SSc and is associated with substantial inpatient morbidity (6).

At the case-report level, chronic intestinal pseudo-obstruction (CIPO) has been described as an uncommon but severe presentation of SSc, sometimes presenting with marked distension, pain, vomiting, and nutritional compromise (7). Mechanical sequelae of colonic dysmotility are also recurrent themes in the literature: fecal bezoar/impaction causing large-bowel obstruction requiring colonoscopic disimpaction (16), chronic megacolon complicated by obstruction (19), and even spontaneous colonic perforation in the context of newly recognized SSc (20). These reports collectively underscore that the “scleroderma colon” phenotype is not benign and may lead to life-threatening complications, particularly when severe constipation, stool retention, or recurrent pseudo-obstruction episodes are present (16, 18–20).

Therapeutically, published experiences also support escalation pathways in refractory pseudo-obstruction-spectrum disease. Octreotide has a physiologic and clinical evidence base in SSc for improving intestinal motility and reducing bacterial overgrowth (21), and combined prokinetic strategies (e.g., octreotide with erythromycin) have been described in scleroderma-associated pseudo-obstruction (15, 17). More recently, immunomodulatory strategies have been reported in select cases of severe CIPO in SSc, including a case treated successfully with intravenous immunoglobulins and rituximab, suggesting that inflammatory or immune-mediated mechanisms may contribute in carefully selected patients (22). Compared with much of the published literature, our case is distinctive for occurring in a young adult male with profound nutritional compromise and colonoscopic evidence of marked hypomotility without a structural lesion, and for improving with targeted therapy aimed at dysmotility-associated symptoms and presumed bacterial overgrowth.

### Limitations and implications

This report is limited by the inherent constraints of a single-case design and by the absence of specialized motility testing (if unavailable) that could further phenotype the dysmotility (neuropathic vs. myopathic patterns) and quantify small-bowel involvement. Nevertheless, the clinical picture and colonoscopic observations provide strong supportive evidence for severe SSc-related lower-GI dysfunction. Clinicians should maintain a high index of suspicion for SSc in patients with unexplained severe dysmotility, rapid weight loss, and multisystem features (Raynaud's phenomenon, digital ulcers, skin tightening), because early recognition enables earlier symptom-directed therapy, nutritional rescue, and surveillance for other organ involvement (2–4).

In conclusion, this case highlights severe SSc-related lower-GI dysmotility presenting early with profound nutritional impact. A structured approach that excludes mechanical obstruction, treats dysmotility and SIBO, and prioritizes nutritional stabilization can lead to meaningful clinical improvement, while vigilance for serious colonic complications remains essential (6, 16, 18–20).

### Clinical takeaways

1. Severe lower- GI dysmotility can be a dominant early manifestation of SSc, even in young males (4).
2. In obstructive- type symptoms, exclude mechanical obstruction but bearing in mind that pseudo-obstruction when there is marked hypomotility or stool retention without a lesion (6).
3. Extra-intestinal features (skin thickening, digital ulcers, microstomia, Raynaud-type episodes) should trigger EULAR/ACR criteria application even when antibodies are atypical.
4. Early management should prioritize nutritional assessment and symptom-focused therapy, including treatment of suspected SIBO when clinically indicated (5, 10, 11).

### Patient perspective

Over the past three years, I experienced progressive unintentional weight loss with worsening abdominal pain, bloating, and changes in bowel habits, which made eating difficult and affected my daily life. Later, I noticed my fingers turning bluish in cold weather and increasing stiffness/skin changes, but I did not initially connect these symptoms. After the diagnosis was explained and treatment was started, I felt clear improvement and began to regain weight.

## Conclusion

This report presents a rare and severe form of systemic sclerosis in a young male patient, in whom early and predominantly lower gastrointestinal dysmotility led to profound nutritional decline. The case highlights that significant gastrointestinal involvement can appear at or near the onset of disease and may dominate the clinical presentation, even before other systemic features become fully established. Marked weight loss, hypoalbuminemia, and colonic hypomotility should therefore raise suspicion for systemic sclerosis in patients with otherwise unexplained gastrointestinal dysmotility, regardless of age or sex. Timely recognition, careful exclusion of mechanical causes, and early initiation of targeted symptomatic and nutritional management are crucial to reducing morbidity and preventing life-threatening complications. This case emphasizes the importance of maintaining clinical vigilance and adopting a multidisciplinary approach when managing gastrointestinal manifestations of systemic sclerosis, particularly in early or atypical presentations.

## Data Availability

The original contributions presented in the study are included in the article/supplementary material, further inquiries can be directed to the corresponding authors.
